# Dynamics of health information-seeking behaviour among older adults with very low incomes in Ghana: a qualitative study

**DOI:** 10.1186/s12889-020-08982-1

**Published:** 2020-06-15

**Authors:** Williams Agyemang-Duah, Francis Arthur-Holmes, Charles Peprah, Dina Adei, Prince Peprah

**Affiliations:** 1 Department of Planning, Kwame Nkrumah University of Science and Technology, Kumasi, Ghana; 2 Independent Researcher, Accra, Ghana; 3 Social Policy Research Centre, University of New South Wales, Sydney, Australia

**Keywords:** Health information-seeking behaviour, Healthcare providers, Older adults with very low incomes, Health policy, Ghana

## Abstract

**Background:**

Exploration of health information-seeking behaviour among older adults with very low incomes is critical in shaping our understanding of how health information is sought in later life. Although studies have focused on health information-seeking behaviour among older people worldwide, subjective views of older adults, especially those with very low incomes in Ghana remain scant. Thus, this study aimed to fill this significant knowledge gap by exploring health information-seeking behaviour among older adults with very low incomes in Ghana.

**Methods:**

In-depth interviews and focus group discussions were conducted with 30 older adults with very low incomes, 15 caregivers and 15 formal healthcare providers in the Atwima Nwabiagya District of Ghana. A thematic analytical framework was used to analyse the data.

**Results:**

The study revealed multiple sources of health information to include healthcare providers, family members, media and friends. The kind of health information sought by older adults with very low incomes consisted of information on diets, causes of chronic non-communicable diseases and medication dosage. The study also identified inadequate knowledge about the benefits of seeking health information, perceived poor attitude of healthcare providers and communication problems as the factors that limit older adults with very low incomes from acquiring health information.

**Conclusion:**

An adequate and reliable source of information is essential to promoting the health of older people. Their inability to secure the right health information could further worsen their health status**.** Thus, the study provides the need for appropriate health policy interventions on the sources and types of health information sought by older adults with very low incomes in Ghana. Healthcare providers are recommended to remain open, friendly and receptive to older people to allow them to seek and obtain health information as they [healthcare providers] constitute the most reliable health information source.

## Background

In recent decades, the rapid ageing of populations, largely due to reductions in fertility and mortality rates, remains a distinctive demographic process [1]. The number of people aged 60+ years is increasing by 3.2% every year, and it is projected to follow a similar trend in the years ahead [2]. Ghana has one of the largest and fastest-growing older populations in sub-Saharan Africa [3, 4]. It is estimated that the proportion of older people (aged 60+) in Ghana will reach about 12% of the general population in 2050 compared with 6.9% in 2015 [3]. According to Biritwum et al. [5], more than half of the vulnerable and older adults with very low incomes in Ghana live in rural areas, where low levels of formal education and income, less access to healthcare, and stark inequalities exist. Older people in this context face burdens of a wide range of disabilities and chronic diseases [6, 7], especially in rural areas where healthcare is widely inaccessible [8].

Conditions associated with ageing such as frailty, disability, chronic diseases, physiological changes, functional limitations, psychological distress, and cognitive decline [9, 10], cause older people to seek treatment and health information to address their ailments and maintain good health and wellbeing. The importance of seeking health information in later life has been highlighted in previous studies [11, 12]. For instance, Chaudhuri et al. [11] asserted that obtaining data about health information-seeking behaviour among older people is important for developing appropriate social and health policies. Feltwel and Rees [12] also emphasised that health information-seeking behaviour has been shown to reduce uncertainty and anxiety of patients. In addition, health information-seeking behaviour results in positive change in patients’ behaviours towards medical treatments [13]. Evidence suggests that health information-seeking behaviour is potentially and independently determined by multifaceted individual demographic, socioeconomic, institutional and systemic factors (such as income, educational level, rurality/urbanity, religion, social support networks, health status, trust, and the political economy of health and healthcare) and the orientations of health system response [11, 13–16]. Meanwhile, sources of health information in descending order of magnitude consist of healthcare providers, pharmacists, friends and relatives, retired community staff, newspapers, internet, television, and radio stations [11]. In most parts of the world, where access to healthcare providers, internet and media outlets are limited, informal information sources, such as friends and family members remain the primary sources of health information for older people [17, 18].

Although studies have focused on the concept of health information-seeking behaviour among older people globally [11, 14, 19], subjective views of older adults with very low incomes on factors that affect their health information-seeking behaviour and sources of health information remain scant in Ghana. That notwithstanding, studies on older adults with very low incomes in Ghana have examined the motivation for utilising private and public healthcare facilities [20], prevalence and patterns of healthcare utilisation [21], barriers to healthcare utilisation [22], healthcare expenditure dynamics [23] and healthcare use predictors [24, 25]. A study in Nigeria has also looked at healthcare-seeking behaviour among older adults [26]. We are however interested in health information-seeking behaviour among older adults with very low incomes in Ghana. Therefore, this study is timely as it explores a disadvantaged group’s health information-seeking behaviour in Ghana to fill a significant knowledge gap in the literature. This study has some potential implications: firstly, understanding health information sources and the kind of health information sought by older adults with very low incomes in Ghana is vital to health actors in their quest to strengthen efforts towards improving older people’s access to quality health information and; lastly, an in-depth exploration of health information-seeking behaviour among older adults with very low incomes is critical to shaping our understanding of how health information is sought in later life.

## Methods

### Study design and context

This paper was extracted from wider ageing, health, lifestyle and health services (AHLHS) study conducted between 1 and 20 June 2018 in the Atwima Nwabiagya District of Ghana. Other aspects of the original research have been reported elsewhere [20–25]. A cross-sectional design involving both quantitative and qualitative strands of research was used for the wider study. Data were drawn on findings from the qualitative section of the original study exploring health information-seeking behaviour among older adults with very low incomes in Ghana. Older adults with very low incomes were defined as persons of 65 years or more experiencing different forms of poverty and receiving financial support from the Livelihood Empowerment Against Poverty (LEAP) programme. The total number of older people in the district was 5430. Of this number, 24.4% were aged between 65 and 69 years, 31.7% were aged 70–74 years, 17.4% were aged 75–79 years and 26.5% were aged 80 years or above. To this end, the LEAP programme criteria helped us to identify older adults with very low incomes for the study so that we could investigate their health information-seeking behaviour in Ghana.

The LEAP programme is a cash transfer intervention sponsored by the Government of Ghana, the World Bank and the United Nations International Children’s Emergency Fund **(**UNICEF). It provides financial protection to people and households that are considered extremely poor [27, 28]. Beneficiaries of the LEAP programme include persons with a disability, older people aged 65 years or more, and orphaned and vulnerable children [27, 29]. They receive a bi-monthly minimum and maximum amount of GH¢ 64.00 and GH¢106.00 (US$13.42–22.23)^1^ respectively [23].

There were 28 health facilities in the district. The majority of the health facilities (53.6%) were owned by private practitioners with 42.9% belonging to the Ghana Health Service (GHS). Also, 3.5% of the health facilities belonged to the Christian Health Association of Ghana (CHAG).

### Participants and recruitment procedures

We conceptualised public health facilities as non-profit healthcare facilities operated, supervised and funded by the government, whereas private healthcare facilities were defined as profit-oriented healthcare facilities owned, operated and funded by private individuals or organisations. Two public health centres (Nkawie Toase and Akropong Health Centres), three private (Afari Community Hospital, Dr. Frimpong Boateng Medical Centre and Mount Sinai Hospital) healthcare facilities and three communities (Kobeng, Amadum-Adankwame, and Offinso Adagya) were purposively selected. We selected these health facilities because they were the most consulted medical facilities by older adults with very low incomes in the district as confirmed by the AHLHS survey [20, 21]. The recruitment of multiple health facilities and communities was also appropriate because the study sought to obtain diverse health information-seeking experiences from different health stakeholders to draw meaningful conclusions. We included three sets of respondents, namely, older adults with very low incomes, caregivers and health professionals. Family members or any other persons providing care for older adults with very low incomes in the study communities were considered as caregivers. However, formal healthcare providers were defined as health professionals, such as doctors and midwives, who have knowledge about the diagnosis or treatment of diseases afflicting people and have worked in the medical field for at least three years preceding the study. The caregivers and health professionals were included in the study because of their key roles in older people’s treatment and health information-seeking as well as in their general health maintenance and promotion [22, 23]. Non-probability techniques, such as purposive and convenience sampling, were used to recruit 60 study participants comprising of 30 older adults with very low incomes, 15 caregivers, and 15 formal healthcare providers.

### Data collection instruments and procedure

Data were obtained through the use of in-depth interviews and focus group discussions (FGDs). These techniques were highly recommendable since the study sought to gain an understanding of the health information-seeking behaviour of older adults with very low incomes through their experiences, views, arguments, and suggestions [30–32]. The techniques enabled the researchers to gain valuable insights into the data provided by the respondents. The interview guides were developed in English but were later translated into Twi (the main dialect in the study area) during interviews since most of the study participants, especially the older adults with very low incomes and caregivers could not read nor write in English. However, for healthcare providers, the interview guide used was not translated into Twi because they were all literates with tertiary level of education. The older adults with very low incomes and caregivers were interviewed at home which was free of interference by any third party. Formal healthcare providers were interviewed in consulting rooms after the end of their usual daily activities. Interviews with older adults with very low incomes lasted between 40 and 45 min whiles that of caregivers and healthcare providers took 30 to 35 min.

Also, we conducted 3 FGDs for only older adults with very low incomes at open places convenient for group interviews. Each group discussion was comprised of 8–10 participants and lasted approximately 90 min. Interviews and FGDs were audio-recorded with informed consent from the study participants [30].

### Ethical approval and consent to participate

Ethical approval was granted by the Committee on Human Research Publication and Ethics (CHRPE), Kwame Nkrumah University of Science and Technology (KNUST), School of Medical Sciences, and Komfo Anokye Teaching Hospital, Kumasi, Ghana (Ref: CHRPE/AP/311/18). The purpose of the study was explained to the study participants before their informed written and verbal consent was obtained. Also, the study participants were assured of strict confidentiality and anonymity of the information they provided. They were further assured that their participation in the study was voluntary. Therefore, they were free to opt-out at any time.

#### Trustworthiness

Trustworthiness in this study was enhanced by emphasising strongly on credibility, transferability, conformability, and dependability through the use of appropriate sampling techniques, such as purposive and convenience sampling. Summaries of the study results were shared among those involved and the participants affirming that the findings reflect their expressed views, feelings, experiences and suggestions. The transcribed interviews and field notes were given to an expert in qualitative research for critical scrutiny to ensure quality control.

### Data analysis

Audio records were transcribed into Twi and later translated into English. Translations were then cross-checked with the recordings and handwritten field notes to ensure quality control and to also identify similar trends and differences in the data. Thematic analysis was used to identify, analyse and report patterns within the data obtained from the field. It aided in organising and describing the data in rich detail [33]. The study results were grouped into themes, and the normative views of the study participants were presented in a form of quotes and excerpts.

## Results

### Background characteristics of participants

Background characteristics of the respondents are presented in Table 1. Table 2 presents the main and sub-themes that were identified during analysis of the data. The specific themes and sub-themes were selected against other important issues that transcend health information-seeking behaviour based on their identified degree of importance to most of the participants during the discussions.

**Table 1:** Characteristics of the study participants

Variable	Category	Category of Respondents (***N*** = 60)		
		Healthcare users (older adults with very low incomes) (***N*** = 30)	Healthcare providers (***N*** = 15)	Caregiver (N = 15)
**Gender**	Male	7	6	–
	Female	23	9	15
**Education**	None	19	–	8
	Basic	8	–	5
	Secondary	3	–	2
	Tertiary	–	15	–
**Religion**	Christianity	27	12	14
	Islam	3	3	1
**Ethnicity**	Akans	25	12	13
	Non-Akans	5	3	2
	Hypertension	15	–	–
	Diabetes	7	–	–
**Medical conditions**	Arthritis	3	–	–
	Asthma	2	–	–
	Stroke	2	–	–
	Depression	1	–	–

**Table 2:** Key themes and associated sub-themes

Main themes	Sub-themes
Sources of health information	**•***Healthcare providers at the health facility*
	**•***Family members*
	**•***Media*
	**•***Friends*
Kind of health information sought	**•***Diet*
	**•***Causes of diseases/illness*
	**•***Medication dosage*
Factors limiting older adults with very low incomes from acquiring health information from healthcare providers	**•***Inadequate knowledge about the benefits of seeking health information*
	**•***Perceived poor attitude of health workers/healthcare providers*
	**•***Communication/language problem*

### Sources of health information

There were four main sources of health information available to older adults with very low incomes interviewed in the Atwima Nwabiagya District. These consisted of healthcare providers, family members, media and friends.

#### Healthcare providers

Most of older adults with very low incomes mentioned that they sought health information from healthcare providers at healthcare facilities, particularly public health centres to either manage their health problems or improve their health after their visit. It was found that talking to healthcare providers for information was not done in isolation but rather in addition to actual healthcare seeking, which is mostly done concurrently. They acknowledged that healthcare providers had been purposely trained to provide healthcare services to people including older people. Hence, healthcare providers were the right source to seek health information. Corroborating these findings, a female older adult with very low income at Kobeng indicated that:

*Mostly I know that the doctors are the right source to seek health information from because they have been trained to do so. Also, I believe that doctors have the knowledge to critically examine me and prescribe medicine for me* (A 73-year female older adult with very low income, in-depth interview).

A male older adult with very low income also noted that:

*Though we ask the doctors and nurses for some information concerning our health, we do not do this when we are not going to seek treatment for certain problems. I think it is time we purposely go there to seek just information and not treatment* (A 66-year male older adult with very low income, in-depth interview)

The healthcare providers admitted that they constituted the right source of health information on healthcare to older people. Further, they emphasised that adequate and reliable sources of information are essential to promoting the health of older people and thus, the inability of older adults with very low incomes to secure the right information could further worsen their health. However, the providers bemoaned that the older people sought information about treatment, but they rarely asked for information concerning their general health status. Some of the providers believed health information-seeking could be done in isolation and not concurrent with the actual treatment. This is evident in what a healthcare provider at Mount Sinai Hospital said:

*An adequate and reliable source of health information is crucial to the health of people. Anytime older people come to the hospital, they come purposely for treatment and few of them try to ask me health information on the causes of diseases, drug intake, and diet. I think they can come around to ask for relevant information about their health and wellbeing (*A 48-year healthcare provider, in-depth interview).

Three caregivers also stated that healthcare providers had information about the medical history of older adults with very low incomes who visited their health facilities. They further argued that since healthcare providers had the necessary information about their patients, they acted as a good source of health information for older adults with very low incomes in Ghana. To this end, one caregiver at Kobeng said that:

*It is advisable and laudable for older people to seek health information from them because doctors are mostly aware of the medical history of older adults with very low incomes who disclose their health problems* (A 44-year caregiver, in-depth interview).

#### Family members

The study revealed that older adults with very low incomes sometimes sought health information from their family members. The older adults with very low incomes interviewed emphasised that they had established a strong bond with their family members (such as children, siblings, and grandchildren), and as such, it was necessary to seek health information from them. Most of the older adults with very low incomes expressed that their family members had been paying for their medical bills, so it seemed right to ask healthcare providers questions about their health. However, family members, who stayed with older relatives, acted as caregivers, and this allowed them to provide their care recipients with needed health information. In line with this, a male older adult with very low income at Offinso Adagya in a FGD had this to say:

*At times, I seek health information from family members because they are closer to me and some of them have been good to me in terms of paying my medical bills* (A 69-year male older adult with very low income, FGD).

Another male older adult with very low income at Kobeng added that:

*I have been seeking health information from my family members (such as children, siblings, and grandchildren) whom I stay with. Without them, it will be very difficult to obtain the right information concerning my health, where I need to seek treatment for my illness and the people I need to obtain treatment from* (An 80-year male older adult with very low income, in-depth interview).

A healthcare provider at Nkawie Toase Government Hospital noted that:

*Some older adults with very low incomes have been seeking health information from their family members because their family members stay with them and know the health conditions they suffer from. This makes it easy for family members to recommend medication for them and cater for their health needs. Yet, older adults with very low incomes should not depend on family members for health information and treatment. There is a cause for alarm if older adults with very low incomes only seek health information from sources where there is no guarantee for their safety* (A 45-year healthcare provider, in-depth interview).

#### Media

The media both print and electronic was identified as one of the sources of health information for older adults with very low incomes. The study participants made it clear that of late, most radio and television stations provide health education. Consequently, the older adults with very low incomes mentioned that they had taken advantage of this new development to source health information from the media, particularly radio and television stations. Though it was seen as very helpful, some older adults with very low incomes declared that they do not have television or radio to gain access to such information. An older adult with very low incomes at Amadum-Adankwame stated that:

*These days, the media has been helping me a lot. They mostly give education on older people's health and this has been very helpful. Even some medical doctors come on radio stations to give a talk on diseases and how to manage it* (A 65-year male older adult with very low incomes, in-depth interview).

Supporting this, a healthcare provider at Mount Sinai Hospital indicated that:

*Recently, older people have been getting health information from the media although we cannot be sure of its quality* (A 52-year healthcare provider, in-depth interview).

#### Friends

A section of older adults with very low incomes (8) indicated that they sourced health information from friends when the need arises. However, it was made known that they did not frequently access information from friends. From the FGD account, older adults with very low incomes sometimes decide to obtain health information from friends upon discussing their illness. For example, a male older adult with very low income at Offinso Adagya said:

*“At times I seek health information from my friends. When I am sick and tell my friends, some suggest to me what needs to be done about my illness. Some even tell what is wrong with me and suggest some traditional medicines to buy and use it” (*A 66-year male older adult with very low income, FGD)*.*

Also, the FGD account revealed that where healthcare providers failed to provide information on diseases being experienced by older adults with very low incomes especially its cause, friends and family members became their important source of health information. Nevertheless, friends and family members turned out to be the only hope of older adults with very low incomes to find the cause of their illness. Consequently, their friends or family members led them to resort to other forms of healthcare such as consulting a traditional medical practitioner or spiritualist/healer for information and treatment.

### Kind of health information sought

For the kind of health information, this study found that older adults with very low incomes sought health information on diet, causes of diseases and medication dosage.

#### Diet

One of the major types of health information sought by older adults with very low incomes was issues concerning their diet. Most of the older adults with very low incomes interviewed mentioned that the kinds of food they eat have a role to play in promoting or worsening their health conditions. They were very conscious of the kind of food they consume. However, healthcare providers stressed that they advised older people to consume a lot of fruits and vegetables and reduce the intake of salty food to improve their health. From the FGD account, several older adults with very low incomes confirmed that they have been following the advice of doctors and nurses on their diet because it could contribute positively to their wellbeing. This is what a male older adult with very low income at Amadum-Adankwame had to say:

*During old age, the kind of food you will eat is very important in promoting health, so I normally ask of the food to eat so that I can always stay healthy. I even heard that some foods can help to reduce high blood pressure at old age* (A 74-year male older adult with very low income, FGD).

Also, healthcare provider at Akropong Health Center stated that:

*The food you eat will determine your health. Most older people including those with very low incomes seek health information on their diets and we also advise them not to eat in the night and must eat more fruits, vegetables and reduce their intake of salt* (A 44-year healthcare provider, in-depth interview).

Besides, one caregiver at Offinso Adagya also stated:

*Anytime, I go to the hospital with my grandfather, he asks the doctor about the kind of food to eat and the food he should not eat. This information has been helpful and has provided me with knowledge as to how I should prepare food for my grandfather who depends on me* (A 50-year caregiver, in-depth interview).

#### Causes of diseases

The study also discovered that older adults with very low incomes sought information about their health on the causes of the diseases or illnesses they suffer from. At their age, most older adults with very low incomes suffer from chronic Non-Communicable Diseases (NCDs) including diabetes, hypertension, arthritis, asthma, stroke and depression. However, several older adults with very low incomes expressed that healthcare providers informed them to frequently resort to positive health behaviour, such as engaging in physical activity, so that they could reduce the incidence of NCDs. Also, healthcare providers indicated that they usually educate older people to practice good health behaviour to stay healthy and live longer. A healthcare provider at Akropong opined that:

*Mostly, I provide health information that will make older adults with very low incomes feel better or promote their health. And I also try to educate them on good health practices and to engage in a good lifestyle***(**A 44-year healthcare provider, in-depth interview)**.**

A healthcare provider at Afari Community Hospital added:

*Okay. Most of the older people seek information for the causes of their chronic conditions such as hypertension and diabetes*. *When older people begin to experience different health problems, they come to us to find out what is wrong with their health* (A 41-year healthcare provider, in-depth interview).

A male older adult with very low income at Kobeng re-affirmed:

*The doctors have informed me that as one grows, the likelihood of developing diseases increases. However, when I go to the hospital, I ask of information about the causes of diseases I suffer from so that I can do my best to minimize the diseases I have* (A 73-year male older adult with very low income, FGD).

A caregiver at Offinso Adagya also reiterated:

*Ageing comes with diseases, so anytime I take my father to the hospital, I seek information about the cause of diseases and provide the necessary action which needs to be taken to prevent certain diseases* (A 50-year caregiver, in-depth interview).

These findings empirically point to the fact that older adults with very low incomes usually sought health information on causes of NCDs at the expense of communicable diseases. For those older adults with very low incomes, who experienced new health conditions, they found it expedient to seek health information to avoid their conditions from worsening. Surprisingly, the older adults with very low incomes interviewed did not mention finding the cause of communicable diseases they suffered from. This was attributed to the fact that some were aware of and had knowledge of communicable diseases (such as tuberculosis and skin diseases) at their age. Some also stated that they could only access health information from healthcare providers when they had been infected with communicable diseases.

#### Medication dosage

Drug intake is very important in the healthcare delivery process. Several older adults with very low incomes indicated that they sought health information on how to take their drugs after the doctor had prescribed medication for them. It was noted by the older adults with very low incomes interviewed that how and when to take a drug based on doctors’ prescription was very important in restoring good health, however, the failure to conform to doctors’ prescription on drug intake could negatively affect their health restoration process. It was made known that good adherence to doctor’s prescription on drugs intake helped to prevent under-dosage or over-dosage of medication. To give more credence to the argument, a female older adult with very low income at Kobeng stated that:

*You see, when to take drugs and how to take it is very important in terms of helping one to restore good health. Mostly, the doctors tell us to follow their prescriptions to avoid over-dosage and under-dosage of drugs* (A 73-year female older adult with very low income, FGD).

Besides, a healthcare provider at Nkawie Toase Government Hospital had this to say:

*Taking drugs is important to us since poor health conditions demand it. If you are hypertensive, you will have to know how to take your drugs well because you will have to take medication every day for the rest of your life. Hence, it is relevant for older people to know how to take their medications* (A 50-year healthcare provider, in-depth interview).

#### Factors limiting older adults with very low incomes from acquiring health information

Although healthcare providers were commended for their role in giving useful health information for older adults with very low incomes and other patients, inadequate knowledge about the benefits of seeking health information, perceived  poor attitude of health workers/healthcare providers, and communication/language problem were pointed out as the main factors that limited older adults with very low incomes from acquiring health information from healthcare providers in Ghana.

### Inadequate knowledge about the benefits of seeking health information

It was observed that older adults with very low incomes did have little knowledge about the importance of accessing health information from healthcare providers, particularly doctors and nurses, at their age. They emphasised that the community address system should be used to educate older adults with very low incomes about their health and the need to seek healthcare from health professionals. However, some felt that they did not see the need to consult doctors for health information since they could purchase medicines at drug stores or pharmacy shops and seek traditional therapy in their communities rather than travel to access health information. For example, a female older adult with very low income at Kobeng said:

*Why should I seek health information from doctors when I can get health information from my friends or relatives and buy medicines at drug stores? I do not need to see a doctor for normal symptoms like headaches, and feeling feverish. For we the people in rural areas, we are not fond of seeking health information from healthcare providers* (A 73-year female older adult with very low income, in-depth interview).

A caregiver at Offinso Adagya also stated:

*Some older adults with very low incomes do not like to go to the hospital to see doctors for information about their health. I always tell the person I am taking care of that she should allow me to take her to the hospital so that she would know what she is suffering from. I think that because they [older adults with very low incomes] do not have enough information on the benefits of seeking health information from healthcare providers, they feel reluctant to see them* (A 40-year caregiver, in-depth interview).

### Perceived  poor attitude of healthcare providers/health workers

The unfriendly attitude of healthcare providers, particularly nurses and non-healthcare providers was identified as a factor that limits older adults with very low incomes from seeking information about their health. Most of the older adults with very low incomes interviewed stressed that healthcare providers should approach their work with professionalism and strengthen their interpersonal skills as they constantly talk to people who come for health information and treatment. From the FGD account, older adults with very low incomes argued that most of the nurses did not accord them with respect and had no time to listen to their health needs. Some mentioned that the doctors at both public and private hospitals did have time for them though they occasionally showed signs of being not ready to attend to their health needs. Interestingly, almost all the participants interviewed shared that the poor attitude of female nurses towards older adults with very low incomes seeking health information was common at public health facilities. However, they argued that the manner in which some nurses talked to them generally prevented them from visiting public hospitals and clinics for health treatment and information. Both the caregivers and older adults with very low incomes emphasised that unfriendly behaviour and disrespectful attitude of most nurses and non-healthcare providers was not common in private health facilities. This is because, despite the economic status of patients who attended the facilities, they were all given equal attention, respect, and care. Supporting the findings, a male older adult with very low income at Amadum-Adankwame had this to say:

*The nurses, particularly those who work at government hospitals talk to me anyhow as if I am a child. Most of the healthcare providers are impatient, rude and not friendly. At our age, the nurses and those who work at the health records unit should treat us with respect because we can give birth to them (*A 68-year male older adult with very low income, in-depth interview*).*

A female older adult with very low income at Kobeng lamented that:

*The way the nurses at public health facilities work is not professional. The nurses are worse when it comes to having time for patients. They are always in a hurry to finish and leave when we ask them questions about our health. Some even tell me to see the doctor for they are not doctors to respond to people’s questions. The attitudes that healthcare providers at government hospitals show to us cannot be found in private hospitals or clinics* (A 73 -year female older adult with very low income, FGD)*.*

### Communication/language problems

Language was mentioned as a factor that acts as a barrier for older adults with very low incomes to seek health information from healthcare providers, particularly doctors. Almost all the older adults with very low incomes interviewed indicated that most of the doctors did not understand the local language (Twi) and could not speak it fluently which inhibited effective communication. They further explained that some of the health professionals speak English only. As a result, they found it difficult to understand what the doctors say and communicate with them. This was in the case of older adults with very low incomes who could not speak English at all. For example, a female older adult with very low income at Offinso Adagya opined that:

*Most of the doctors cannot speak Twi fluently so it is difficult for us to communicate with those who are not Akans. I cannot speak the English Language too. Because of the way they speak Twi, it is hard to comprehend what they say* (A 70-year female older adult with very low income, in-depth interview).

Another female older adult with very low income at Kobeng also noted that:

*I remember one occasion I went to the hospital to find out why I usually get severe headaches and fever. I could not understand what the doctor was saying because his Twi was not good. I tried my best to understand it. But he made it hard for me because he was speaking in English Language and Twi. I think that it is best for health institutions to send nurses and doctors to districts where they can speak their local language* (A 78-year female older adult with very low income, FGD).

## Discussion

This study explored the health information-seeking behaviour among older adults with very low incomes in Ghana using empirical evidence from the Atwima Nwabiagya District. Health information-seeking behaviour was assessed in three areas: sources of health information, kind of health information sought by the older adults with very low incomes, and factors that limit them from acquiring health information from healthcare providers. We found that sources of health information by older adults with very low incomes included healthcare providers, family members, media and friends. This finding is in line with previous research. For example, Chaudhuri et al.’s [11] study in Washington State revealed sources of health information to comprise of healthcare practitioners, family members, friends, and media. Redmond et al. [34] assert that people generally depend on mass media (print, TV, Internet, etc), healthcare providers and interpersonal factors (such as nonprofessional social networks consisting of friends, family members, and community organisations) for health information. This study provides evidence to suggest that healthcare providers constitute the most reliable source of health information for older adults with very low incomes. However, seeking health information from healthcare providers by the study participants was not done in isolation from actual healthcare seeking but in a concurrent manner. Thus, seeking health information from healthcare providers is inextricably linked to actual healthcare seeking. Older adults with very low incomes did not visit health facilities purposely to seek for information on their health but rather ask for information in addition to actual healthcare seeking. Also, older people rely on their social support networks, such as family members and friends, for immediate health information [21, 35]. Our findings suggest that older people’s decision to seek health information is influenced by the availability and accessibility of social support networks coupled with healthcare providers. In Ghana, community-dwelling older adults with very low incomes may be more motivated to seek health information with the availability of functioning social support networks and accessible healthcare providers.

Our findings on the media as a source of health information are further elaborated. In most Ghanaian communities, the spread of media outlets in a form of diverse radio stations, newspapers, television telecasts and information centres has emerged and contributed greatly to the sharing of diverse health information for many people including older adults with very low incomes [36–38]. Thus, older adults with very low incomes rely on community radio stations and information centres for health information. We comment that frequent access to health information on radio stations could have potential implications on health information-seeking behaviour among older adults with very low incomes. However, empirical evidence suggests that seeking health information through radio stations has a higher tendency of deepening individuals’ knowledge on health information and subsequently changing their health behaviour [39].

An important finding was that most older adults with very low incomes sought information on their diet, drug intake and causes of diseases they suffer from. This finding is important because older adults with very low incomes, especially those in rural communities are more concerned with their daily livelihood activities than spending time to obtain information on their diets and the causes of their health problems. However, the types of health information sought by older adults with very low incomes are crucial to improving their health status. For instance, knowing the causes of diseases older adults with very low incomes suffer from and the details of their diets are fundamental to helping them adopt an appropriate lifestyle and healthy behaviour, such as engaging in physical activity and consuming fruits and vegetables to promote their health [40–42]. The study revealed that most of the older adults with very low incomes sought health information about NCDs. The reason for this is likely to be twofold; they have greater experience of NCDs (such as hypertension, diabetes, arthritis, asthma, stroke and depression) and the causes of NCDs are by their nature, less clear. Based on these reasons, health stakeholders ought to provide education for older people on the causes of NCDs to deepen their knowledge about it.

We found that some older adults with very low incomes did not have sufficient knowledge about the benefits and relevance of seeking health information from healthcare providers, especially doctors and nurses. This was attributed to three reasons: firstly, they saw no need to consult doctors for information on their health; secondly, they could purchase medicines at drug stores or pharmacy shops and; finally, they could use traditional therapy in their communities rather than travel to access healthcare and seek health information from doctors. The findings suggest that not having sufficient knowledge about the importance of acquiring health information from healthcare professionals may prevent one from receiving the necessary information to carry out medical instructions. While many older adults with very low incomes desired to obtain health information at healthcare centres, the perceived poor attitude of most nurses, particularly those at public health facilities prevented some from seeking health information. Our findings are therefore consistent with previous studies. For example, Van Rooy et al. [43] found that some nurses at public health facilities are rude and impatient. The possible reasons why healthcare providers may be perceived as rude by older adults with very low incomes are further discussed. Older adults due to their conditions may expect healthcare providers to treat them with much care, respect and attention. In the absence of this, they may perceive the healthcare providers as being rude. Also, most of these older adults with very low incomes may think that the healthcare providers can either be their children or grandchildren, hence they expect them to communicate in a respectful manner. In the Ghanaian society where people attach dignity to older people, any action that seems inconsistent with the acceptable social behaviour may be deemed rude and disrespectful.

As revealed by the study, most of the older adults with very low incomes interviewed had no formal education and could only speak their local language (Twi), therefore serving as a hindrance to seeking health information. In light of this, healthcare providers found it difficult to know the health needs of older adults with very low incomes when they did not understand their local language and what they were saying. However, the inability of healthcare providers, especially doctors to communicate in the local language of health information seekers affects the utilisation of healthcare and healthcare processes [44]. Besides, older adults with very low incomes need to feel comfortable to express themselves and communicate their symptoms of diseases to healthcare providers. Thus, stakeholders, particularly the Ministry of Health and Ghana Health Service should consider the socio-cultural background of health professionals when posting them to a health facility. Studies have shown that older people do not utilise healthcare and seek health information because of the use of the English Language as a medium of communication in most healthcare facilities [11]. Indeed, language differences serve as a limitation to seeking health information from healthcare providers [43]. Language differences resulting in ineffective communication between older adults with very low incomes and healthcare providers suggest that older adults with very low incomes would not be able to articulate their health information needs to the health professionals. Empirical evidence indicates that poor communication often leads to lack of understanding of the health problem, which is likely to have a negative effect on health [information] outcomes [44]. We, therefore, recommend that there should be recruitment of language translators into the Ghanaian healthcare delivery system in order to ensure effective communication between healthcare providers and older adults with very low incomes [20, 22]. In addition, we encourage caregivers to accompany older people to access healthcare services – health information – from healthcare providers [22].

To the best of our knowledge, this is one of the first studies in Ghana to explore health information-seeking behaviour from the perspective of older adults with very low incomes, healthcare providers and caregivers in the Atwima Nwabiagya District. The specific contribution of this study to literature is that inadequate knowledge about the benefits of seeking health information, poor attitude of healthcare providers/health workers, and communication problems limit older adults with very low incomes from acquiring health information. Also, older adults with very low incomes focus more on seeking NCDs’ health-related information. This study therefore, advances knowledge in relation to older adults with very low incomes and their health information-seeking behaviour. Despite these strengths, the findings of this study could not be generalised due to the use of non-probability sampling techniques with a limited sample size. However, we were interested in seeking an in-depth analysis of health information-seeking behaviour rather than the trends and patterns of it. Also, the study did not provide a detailed analysis of socio-economic, demographic and health-related factors influencing healthcare information-seeking behaviour among older adults with very low incomes in Ghana due to its qualitative nature. For this reason, rigorous quantitative research is needed to bring to bear socio-demographic and health-related factors influencing health information-seeking behaviour among older adults with very low incomes in Ghana.

### Implications for policy and practice

This study is an attempt to better understand the pathways and potential dynamics of health information-seeking options among older adults with very low incomes. This is an important subject given the complexities of diverse socio-economic and health circumstances of ageing adults, particularly in a lower-income setting. This study, therefore, offers several potential implications for policy and practice. The findings of the study suggest the need for health actors to provide relevant information on health-seeking behaviour among older adults with very low incomes to inform reliable and quality health information in later life. In addition, public health and policy frameworks intended to improve people’s access to health information should also adopt measures to address barriers to health information-seeking behaviour among older adults with very low incomes. Such interventions to address the barriers to health information –seeking behaviour among the study participants may consider the following suggestions. In the first place, frequent and periodic education on the importance of health information by regional and municipal health directorates would help to deepen the knowledge of older people on health information. Secondly, recruitment of language translators into healthcare facilities by relevant health stakeholders would help to counter communication or language problems associated with seeking health information. Thirdly, periodic attitudinal change programmes for healthcare providers through conferences, workshops and seminars are critical to addressing attitudinal barriers to seeking health information among older adults with very low incomes. As a sequel to the aforementioned points, healthcare providers should remain open, friendly and receptive to older people to allow them to seek and obtain health information as they [healthcare providers] constitute the reliable health information source. These strategies would be helpful in terms of reducing health information-seeking gaps among older adults with very low incomes in Ghana.

## Conclusion

This study has presented evidence on the dynamics of health information-seeking behaviour among older adults with very low incomes in Ghana. The findings indicate that older adults with very low incomes rely on their social networks, healthcare providers and the media for health information. However, the availability and accessibility of social support networks may influence health information-seeking behaviour of older adults with very low incomes. Moreover, older adults with very low incomes did not seek health information from healthcare providers in isolation from actual healthcare seeking but in a concurrent manner. While older adults with very low incomes mostly sought information on diets, drug intake and causes of diseases, factors such as inadequate knowledge about the benefits of seeking health information, perceived  poor attitude of healthcare providers and communication/language problems remain barriers to their health information-seeking behaviour. Thus, social strategies that seek to improve access to health information, especially among older adults with very low incomes in Ghana are required.

## Data Availability

The datasets used and/or analysed during the current study are available from the corresponding author on reasonable request.

## Abbreviations

AHLHS: Aging, Health, Lifestyle and Health Services Survey
CHAG: Christian Health Association of Ghana
CHRPE: Committee on Human Research Publication and Ethics
GHS: Ghana Health Service
LEAP: Livelihood Empowerment Against Poverty
NCDs: Non-Communicable Diseases

## Footnotes

1 Exchange as at June 2018 (GH¢ 4.77 = US$1.00)
